# Mitigating low-temperature stress in alfalfa by postponing phosphorus application and remodeling of antioxidant activities and carbon-nitrogen metabolism

**DOI:** 10.3389/fpls.2025.1550026

**Published:** 2025-02-18

**Authors:** Hui Xu, Liying Xu, Muhammad Ahmad Hassan

**Affiliations:** ^1^ College of Landscape and Horticulture, Wuhu Institute of Technology, Wuhu, China; ^2^ College of Resource and Environment, Anhui Agricultural University, Hefei, China

**Keywords:** *Medicago sativa* L., postponing phosphorus application, low-temperature stress, antioxidant properties, carbon-nitrogen metabolism

## Abstract

Low-temperature stress has become a major limiting factor for the sustainable production of forage crops and animal husbandry. This experimental study evaluated the effects of optimizing phosphorus application on the antioxidant properties and carbon-nitrogen metabolism physiology of alfalfa (*Medicago sativa* L.) under LT stress, aiming to provide a reference for efficient stress-resistant alfalfa production. In this study, the LT tolerant cultivar ‘Caoyuan’ (CY) and LT sensitive cultivar ‘Xinmu’ (XM) were used as plant materials, and the physiological changes of alfalfa plants under natural temperature (NT) and LT were compared under traditional phosphorus application (R1) and postponing phosphorus application (R2) treatments. The results showed that LT stress increased the accumulation of malondialdehyde (MDA) in alfalfa plants and inhibited root activity, carbon metabolism, and photosynthesis in both cultivars. The negative impacts of LT are more prevalent in XM than in CY. The postponing phosphorus application treatments enhanced root vitality as compared to the traditional phosphorus application treatments and accumulated more soluble sugar (5.6-11.2%), sucrose (8.5-14.0%), proline (7.5-11.7%), and soluble protein (8.3-11.7%) by increasing the enzyme activities related to carbon-nitrogen metabolism. Under postponing phosphorus application treatments, the enzymatic activities of antioxidants and regulation of osmotic sub-stances significantly increased in the leaves, MDA contents were decreased by 4.6-7.6%, and chlorophyll contents were increased by 4.8-8.6%, the net photosynthetic rate in alfalfa leaves increased by 5.1-7.5%. Besides, plant dry weight, root dry weight, and plant phosphorus concentration increased by 5.8-16.9%, 7.8-21.0%, and 5.1-9.9% under postponing phosphorus application treatments. In summary, split-phosphorus fertilization improved the nutrient absorption capacity of alfalfa roots compared to traditional phosphorus application treatments under LT stress. Moreover, it improved the carbon-nitrogen metabolism physiology and photosynthetic production capacity of the alfalfa plants, thus reducing the adverse effects of LT stress on the growth and development of alfalfa.

## Introduction

Climate change, food security, and population growth are key challenges of the 21st century (Premanandh, 2011). In the context of global climate change, frequent abiotic stresses, such as high and low temperatures, significantly challenge agricultural safety and production (Raza et al., 2019). Alfalfa (*Medicago sativa* L.) is a perennial leguminous forage with high yield and quality (Feng et al., 2022). It has the advantages of good palatability, rich nutrition, and strong adaptability, making it also one of the most cultivated forages in the world (Li et al., 2020a; Suwignyo et al., 2023; Yan et al., 2024). Most of China is in the monsoon climatic zone, which often encounters low-temperature (LT) stress during the winter and spring seasons and causes a significant reduction in crop productivity (Xu et al., 2023b). Therefore, LT stress has become an important factor restricting the sustainable production of alfalfa in China.

Under LT stress, alfalfa plant cells undergo an intensified membrane peroxidation process, resulting in significant oxidative damage to the membrane system and producing malondialdehyde (MDA). This oxidative breakdown product damages the biofilm (Mittler et al., 2022; Zhao et al., 2023). Alfalfa plants can scavenge the reactive oxygen species (ROS) through the activities of antioxidant enzymes (i.e., superoxide dismutase-SOD, peroxidase-POD, and catalase-CAT) for maintaining the cellular stability of the membrane system under LT stress (Liu et al., 2019). Moreover, carbon and nitrogen metabolism, the two most fundamental metabolic processes in plants, are also affected by LT stress (Nunes-Nesi et al., 2010; Yang et al., 2023). In alfalfa, leaves and roots are vital organs for photosynthesis and nutrient absorption and are susceptible to LT stresses. LT stress destroys the membranous structure of alfalfa’s source-sink organs, and excessive ROS accumulation causes severe damage to the photosystem (Allen and Ort, 2001; Liu et al., 2019). This disturbance in the physiological process reduces the assimilates’ accumulation, transportation, and distribution, thus affecting alfalfa’s normal growth and development (Rahman et al., 2024). In addition, the extreme LT stress led to the failure of alfalfa to remain greenish, resulting in a decline in the yield and quality of alfalfa in successive years; it hampered the sustainable development of the forage and industry (Sun et al., 2022). Therefore, there is an urgent need to explore reasonable cultivation models to reduce the negative impacts of LT stress on optimal alfalfa growth.

The production of alfalfa is influenced not only by climatic conditions but also by soil nutrition, which is crucial for normal growth and functioning (Kong et al., 2020). In modern crop cultivation, phosphorus is vital for plant metabolism and the 2nd most essential nutrient after nitrogen (Wang et al., 2021). It plays a pivotal role in plant growth and development, as it is a significant component of plant structures such as adenosine triphosphate (ATP), phospholipids, and nucleotides; it also exists in the form of phosphate groups in various synthesis pathways and is actively involved in crucial metabolic processes, such as respiration, photosynthesis, and synthesis of energy molecules (Lambers, 2022; Poirier et al., 2022). A recent study showed that only 12.6% phosphorus was taken up by plants, 67.2% of inorganic phosphorus fertilizer was stored in soils, and 4.4% was lost through runoff and leaching (Luo et al., 2024). Traditional practices of phosphorus fertilization (i.e., top-dressing at the time of sowing) resulted in leaching and readily fixation of phosphorus with soil particles; hence, poor phosphorus-utilization efficiency was observed in crop plants (Shen et al., 2011; Veneklaas et al., 2012). Since alfalfa is a phosphorus-loving crop, phosphorus nutrition plays a significant role in its growth metabolism and nutrient accumulation (Han et al., 2022). Previous studies have shown that optimizing water and fertilizer management enhances water and nutrient use efficiency by adjusting root morphology and structure, thereby reducing the impact of LT stress on the growth and development of alfalfa plants (Adhikari et al., 2022; Li et al., 2022). It has been revealed that the LT tolerance of alfalfa plants is regulated by the accumulation of osmotic regulatory substances and the activities of antioxidant enzymes (Nunes-Nesi et al., 2010; Zhang et al., 2022). Moreover, reasonable depth of phosphorus fertilizer application improves alfalfa’s LT tolerance by enhancing antioxidant enzyme activities and resulting in increased biomass (Xia et al., 2022). It is reported that optimizing phosphorus application promotes photosynthesis in the leaves of wheat plants, facilitating phosphorus accumulation, thereby improving wheat grain yield under LT stress (Xia et al., 2023, 2024). Therefore, optimizing phosphorus application is significant for enhancing the LT tolerance and quality improvement in alfalfa. However, the underlying mechanism of postponing phosphorus application in reducing the detrimental impacts of LT stress in alfalfa by improving the related physiological characteristics of source and sink organs remains unclear.

In this research study, pot experimentation was carried out to investigate the effects of postponing phosphorus application on the antioxidant properties and physiology of carbon and nitrogen (C & N) metabolism in the source-sink organs of two alfalfa cultivars under LT stress. Our primary objectives are to (1) analyze and quantify the impacts of split-phosphorus application on the antioxidant system in alfalfa leaves and roots under LT stress; (2) elucidate the underlying mechanism of postponing phosphorus application on osmoregulation and carbon-nitrogen metabolism in alfalfa leaves and roots under LT stress; and (3) explore the positive impacts of postponing phosphorus application on the photosynthetic activity in the leaves of alfalfa under LT-stress. This study aimed to establish the theoretical and practical support for high-yield and stress-resistant cultivation of alfalfa in harsh climatic conditions.

## Materials and methods

### Plant material and growth conditions

**Figure 1:**
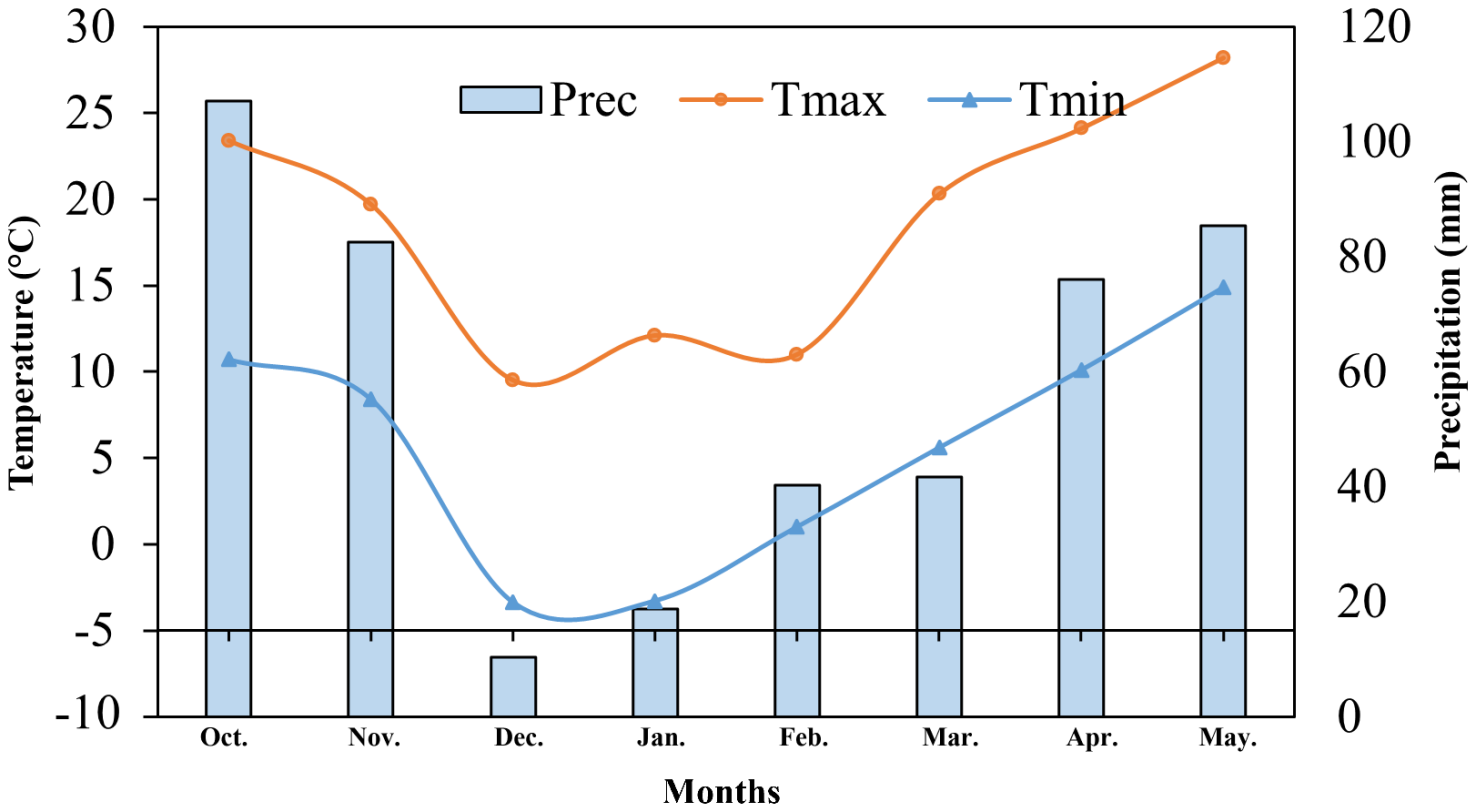
Climatic conditions during the course of experimentation has been illustrated.

This experiment was conducted in a controlled phytotron chamber with a completely randomized design (CRD) in a split arrangement. Two alfalfa cultivars with variable temperature susceptibility, LT tolerant cultivar ‘Caoyuan’ (CY) and LT sensitive cultivar ‘Xinmu’ (XM). The experiment was conducted from October 2022 to May 2023 at the Agricultural Cultivation Garden of Anhui Agricultural University. Climatic conditions regarding temperature (max. & min.) and precipitation have been exhibited in **Figure 1**. The soil type was yellow-brown, with 17.0 g·kg^-1^ organic matter contents, nitrogen (N) of 125.4 mg·kg^-1^, phosphorus (P) of 25.1 mg·kg^-1^, and potassium (K) of 144.2 mg·kg^-1^. Half a month before the experiment, the soil was filled into the pots with drainage holes at the bottom (PVC pots having a diameter of 26 cm and height of 35 cm), with 8 kg of soil per pot. 2.0 g of urea and 2.0 g of potassium sulfate were applied in each pot as a basal fertilizer dose before sowing. Two phosphorus application treatments were applied, including (i) a traditional phosphorus application treatment (R1) in which 2.0 g of diammonium phosphate, a full dose of phosphorus, was applied as a basal dose; (ii) postponing phosphorus application treatment (R2) in which 1.0 g of diammonium phosphate was applied before sowing as a basal dose and remaining top dressed in early February as split-phosphorus dose.

Alfalfa seeds of good viability, high germination rate (up to 95%), and uniform size were chosen for sowing. In each pot, 5 seeds were sown; after germination, 3 plants were maintained by thinning. With its higher altitude, this region is prone to late spring coldness in the second half of March each year (Su et al., 2022), and at this time, alfalfa is in its budding stage. Therefore, in this experiment, pots with uniformly grown plants were moved to an ultra-LT artificial climate chamber on the 15th of March and set an LT treatment of -4°C from 1:00 to 5:00 am. In control treatment (10°C), alfalfa plants were kept in the field conditions (**Table 1**). Each treatment consisted of 20 pots, and there was a total of 160 pots for the two cultivars. After the treatments of LT, the pots of alfalfa were shifted back into the field conditions and underwent sampling, preservation, and measurement of photosynthetic parameters.

**Table 1:** Experimental treatments.

Cultivars	Phosphorus application	Temperature treatments	
CY	R1	NT	LT
	R2	NT	LT
XM	R1	NT	LT
	R2	NT	LT

Here, R1, R2, NT, and LT refer to traditional phosphorus application, postponed-phosphorus application, normal temperature, and low temperature, respectively.

### Sampling and measurements

Immediately after undergoing through LT stress, 15 pots were randomly selected from each treatment, and their leaves with suitable size, complete structure, and no insect damage/broken insects in the middle of the upper one-third and roots were sampled. Sampled alfalfa leaves and roots were immersed in liquid nitrogen, wrapped in aluminum foil, and stored in an ultra-low-temperature freezer at -80°C for later physiological measurements, and 8 replications for each treatment. Photosynthetic parameters and relative chlorophyll content were measured with intact leaves in the middle of the upper one-third of the leaf position. Measure the dry weight and phosphorus content of alfalfa plants and roots at maturity stage.

### Antioxidant enzyme activity and MDA contents

Sampled alfalfa leaves and roots were stored in the ultra-LT freezer and later were used to compute the enzymatic activities of antioxidants (POD, SOD, CAT) and contents of MDA. A 0.1g sample of leaves and roots was crushed and grounded for each measurement. It was repeated three times for each parameter. The enzymatic activities of SOD, POD, and CAT in the leaves and roots were measured using the NBT photo-reduction, guaiacol, and UV absorption method, respectively. The contents of MDA were calculated using the thio-barbituric acid method (Li et al., 2023).

### Osmotic adjustment substances

The determination of osmotic adjustment substances primarily includes soluble sugar (SS), sucrose (SUC), soluble protein (SP), and proline (Pro) contents. A 0.1g sample of leaves and roots was crushed and grounded for each measurement. It was repeated three times for each parameter. The SS contents were determined by anthrone colorimetry, and the resorcinol method was used to determine the SUC contents (Jiang et al., 2022). The SP contents were determined using the G-250 Coomassie blue colorimetric method, and the Pro contents were measured using the sulfosalicylic acid method (La et al., 2019; Zhang et al., 2020).

### Root activity and acid phosphatase activity

The TTC reduction method was used to determine alfalfa’s root activity (Symanowicz et al., 2021). The activity of acid phosphatase (ACP, a key enzyme of phosphorus metabolism) in alfalfa roots was determined using the Solarbio kit (produced by Beijing Solarbio Technology Co., Ltd.). The specific method for determining ACP was carried out according to the kit instructions. The plate reader (Thermo Multiskan FC) was used to compute the ACP activity. There were 3 repetitions in each treatment, and the average value was used for result analysis.

### Carbon and nitrogen metabolism-related enzyme activity

The activities of carbon metabolism-related enzymes (sucrose synthase and sucrose phosphate synthase, i.e., SUS and SPS) and nitrogen metabolism-related enzymes (nitrate reductase and glutamine synthetase, i.e., NR, GS) in leaves of alfalfa were measured using the Solarbio kit and the plate reader (Wang et al., 2021). The specific measurement method followed the instructions provided in the kit. There were 3 repetitions in each treatment, and the average value was used for result analysis.

### Chlorophyll content and SPAD value

Chlorophyll (Chl) content in alfalfa leaves was measured using ethanol extraction. Additionally, after the LT stress, alfalfa leaves’ relative chlorophyll contents (SPAD value) were measured using a SPAD-502 chlorophyll meter, with 3 repetitions (Chen et al., 2018; Kalaji et al., 2017).

### Photosynthetic parameters

After LT stress, the photosynthetic parameters of intact alfalfa leaves were measured using a Li-6400 portable photosynthesis system analyzer produced by LI-COR (USA) with 3 repetitions (Shibaeva et al., 2020). The photosynthetic parameters include net photosynthetic rate (Pn), stomatal conductance (Gs), intercellular CO_2_ concentration (Ci), and transpiration rate (Tr).

### Plant root dry weight and phosphorus concentration

At the maturity stage of alfalfa, 3 pots of plants were selected for each treatment. The plants were cut with scissors 2 cm above the soil surface and up-rooted below part and then cleaned it thoroughly via tap-water washing. Then all samples were wrapped in polythene bags and placed in an oven for 30 minutes for a constant temperature of 100°C. After drying of samples were dehydrated to a constant weight at 75°C. Upon reaching constant weight, all samples were weighed using electric weighing balance. After weighing, used a ball mill (MM400, Retsch Company, Arzberg, Germany) to grind the plants and roots, and use the H_2_SO_4_-H_2_O_2_ method to detect the phosphorus concentration of alfalfa plants (Xu et al., 2023a).

### Statistical analysis and plotting

Data were organized and charted using Microsoft Excel 2019 and Origin 2021. Data analysis was performed using SPSS 19.0 software, and the significance of differences between treatments was tested using the least significant-difference (LSD) technique (*p* = 0.05).

## Results

### Plant dry weight and phosphorus concentrations

Effects of split-phosphorus application on plant dry weight, root dry weight, and plant phosphorus concentration in alfalfa under low-temperature stress are shown in **Table 2**. The plant, root dry weight and plant phosphorus concentration of both cultivars, CY and XM, were decreased after LT stress. Following the split-phosphorus application treatments, they increased to varying degrees. The plant dry weight, root dry weight, and plant phosphorus concentration of CY and XM decreased by 12.0-20.9% and 26.1-36.7%, 5.6-16.6% and 26.7-39.4%, 0-4.5% and 5.0-13.6%, respectively, under LT treatments (R1LT, R2LT). When compared to traditional phosphorus application treatments (R1NT, R1LT), the postponing phosphorus application treatments (R2NT, R2LT) increased plant dry weight, root dry weight and plant phosphorus concentration of CY and XM cultivars by 7.1-12.0% and 5.8-16.9%, 7.8-13.2% and 9.4-21.0%, 5.1-6.3% and 6.1-9.9%, respectively. Furthermore, phosphorus mode had an extremely significant effect on plant dry weight and root dry weight (*P* < 0.01), and a significant effect on plant phosphorus concentration (*P* < 0.05). In contrast, temperature treatment had an extremely significant effect on plant dry weight, root dry weight, and plant phosphorus concentration.

**Table 2:** Effects of split-phosphorus application on plant dry weight, root dry weight, and plant phosphorus concentration of alfalfa under low-temperature stress.

Cultivar	Phosphorus mode	Temperature treatment	Plant dry weight (g pot^−1^)	Root dry weight (g pot^−1^)	Phosphorus concentration (%)
CY	R1	NT	11.70 ± 0.42a	1.52 ± 0.03ab	18.4 ± 0.9a
		LT	9.26 ± 0.25b	1.27 ± 0.06b	17.6 ± 0.7a
	R2	NT	12.54 ± 0.34a	1.64 ± 0.12a	19.4 ± 0.7a
		LT	10.30 ± 0.19b	1.44 ± 0.06ab	18.7 ± 0.7a
XM	R1	NT	13.05 ± 0.42a	1.61 ± 0.08a	20.1 ± 1.0ab
		LT	8.26 ± 0.29c	0.98 ± 0.10c	17.4 ± 0.7b
	R2	NT	13.81 ± 0.45a	1.76 ± 0.09a	21.4 ± 0.8a
		LT	9.65 ± 0.28b	1.18 ± 0.08b	19.1 ± 0.4ab
*F*-value	*F*-_Cultivar(C)_		1.472	2.549	4.478
	*F*-_Phosphorus mode(P)_		25.140**	9.012**	6.769*
	*F*-_Temperature treatment(T)_		289.225**	60.982**	11.322**
	*F-*_C×P_		0.119	0.108	0.203
	*F*-_C×T_		28.358**	12.465**	3.13
	*F*-_P×T_		1.104	0.222	0.097
	*F*-_C×P×T_		0.287	0.001	0.03

Here, R1, R2, NT, and LT refer to traditional phosphorus application, split-phosphorus application, normal temperature, and low temperature, respectively. C, P, T, C × P, C × T, P × T, and C × P × T refer to cultivar, phosphorus mode, temperature treatment, the interaction of cultivar with phosphorus mode, the interaction of cultivar with temperature treatment, the interaction of phosphorus mode with temperature treatment, and the interaction of cultivar, phosphorus mode with temperature treatment, respectively. * and ** indicate significant differences at the 0.05 and 0.01 levels, respectively. The data mean ± *SE* (*n* = 3). Lowercase letters following the data within the same column refer to significant differences (*P* < 0.05).

### Antioxidant enzymes activities and MDA contents in roots

**Figure 2:**
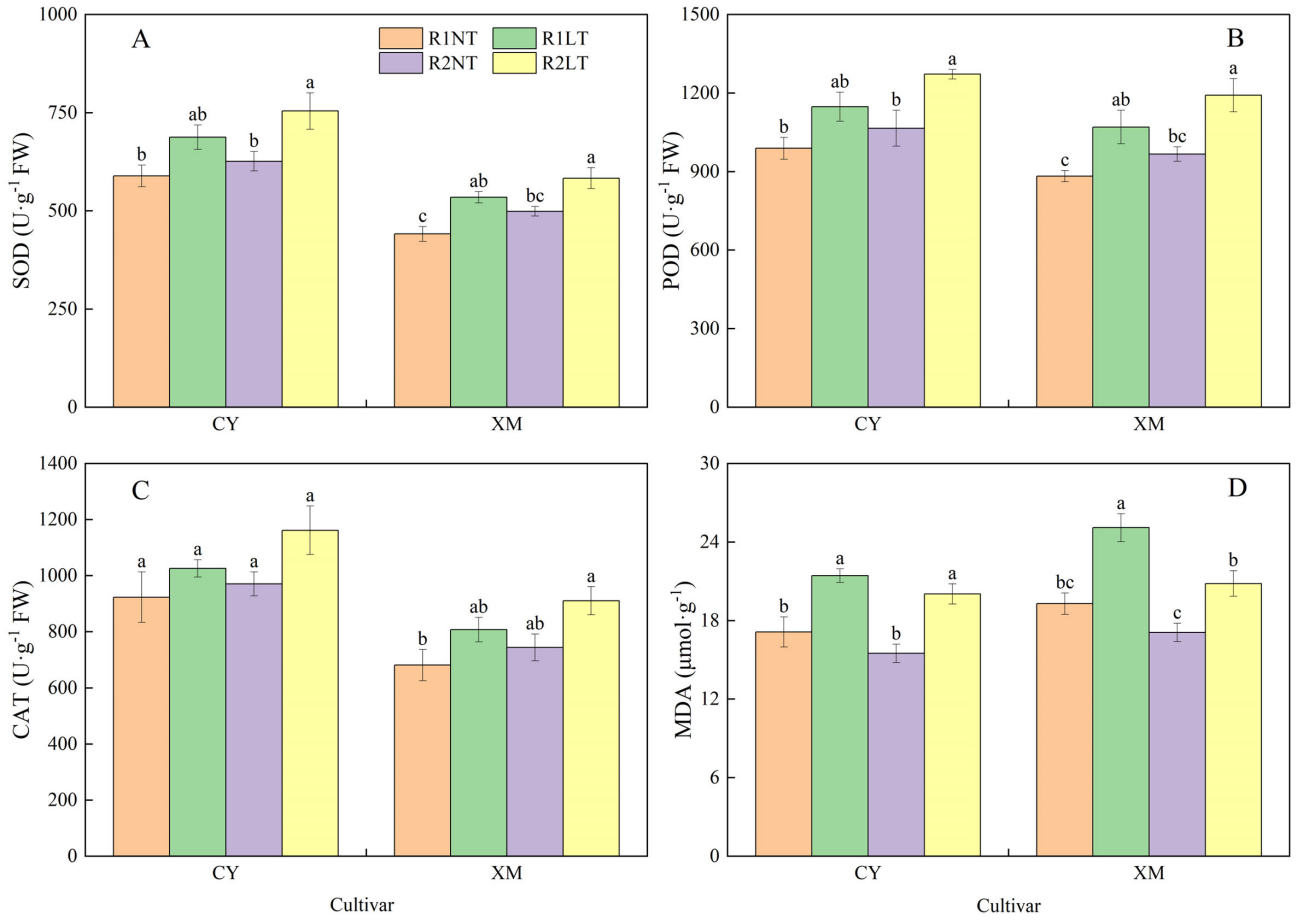
Effects of split-phosphorus application on the enzymatic activities of antioxidants and MDA constituents in alfalfa roots under low-temperature stress. **(A)** Superoxide dismutase (SOD) activities in alfalfa roots, **(B)** Peroxidase (POD) activities in alfalfa roots, **(C)** Catalase (CAT) activities in alfalfa roots, and **(D)** Malondialdehyde (MDA) contents in alfalfa roots. R1, R2, NT, and LT refer to traditional phosphorus application, split-phosphorus application, normal temperature, and low temperature, respectively. The data mean ± SE (*n* = 3). Lowercase letters refer to significant differences between the treatments (*P* < 0.05). Whiskers above the bars indicate the standard error.

As illustrated in **Figure 2**, the activities of SOD, POD, and CAT, as well as MDA contents, in the roots of both cultivars, CY and XM, were increased after exposure to LT stress. Following the split-phosphorus application treatments, the activities of SOD, POD, and CAT showed an upward trend, whereas MDA contents were decreased. In particular, the roots of CY and XM cultivars showed elevated SOD, POD, and CAT activities by 16.8-28.1% and 21qq.1-32.2%, 16.1-28.6% 21.3-35.0%, 11.1-25.8%, and 18.6-33.7%, respectively, under LT treatments. Concurrently, MDA contents in the roots of CY and XM cultivars increased by 16.9-25.1% and 7.9-30.1% following LT treatment. When compared to traditional phosphorus application treatments, the postponing phosphorus application treatments exhibited an increasing trend for SOD, POD, and CAT activities in the roots of CY and XM cultivars by 6.3-9.7% and 9.1-13.1%, 7.8-10.8% and 9.6-11.4%, 5.1-13.3% and 9.3-12.7%, respectively. Additionally, MDA contents in the roots of both CY and XM were reduced by 6.5-9.5% and 11.4-17.0%, respectively, under postponing phosphorus application treatments, and observed a significant reduction in the MDA contents of R2LT treatment of XM compared to the R1LT treatment of XM. Cultivar and temperature had an extremely significant effect on antioxidant enzyme activities and MDA contents in roots (*P* < 0.01), whereas phosphorus mode had no significant effect on CAT activities (**Table 3**).

**Table 3:** Three-way ANOVA analysis for the effects of split-phosphorus application on antioxidant enzymes activities, MDA contents, osmotic regulatory substances, root activity, and ACP activity in alfalfa roots under low-temperature stress, and the interactive effect.

F-value	SOD	POD	CAT	MDA	SS	SUC	SP	Pro	RA	ACP
C	75.095**	7.607*	28.603**	12.522**	3.18	2.296	1.627	2.714	8.420*	0.605
P	9.259**	9.565**	3.97	16.671**	3.909	5.753*	5.358*	5.465*	3.263	4.156
T	34.216**	35.007**	11.164**	62.314**	10.603**	14.585**	17.188**	17.034**	32.305**	66.020**
C×P	0.001	0.002	0.01	2.18	0.007	0.012	0.102	0.017	0.011	0.074
C×T	0.503	0.13	0.00	0.09	0.464	0.434	0.275	0.21	0.373	2.371
P×T	0.087	0.415	0.53	0.62	0.271	0.308	0.011	0	0.049	0.208
C×P×T	0.308	0.006	0.08	0.98	0.051	0.039	0.007	0.016	0.011	0.057

Here, SOD, POD, CAT, MDA, SS, SUC, SP, Pro, RA, and ACP refer to superoxide dismutase, peroxidase, catalase, malondialdehyde, soluble sugar, sucrose, soluble protein, proline, root activity, and acid phosphatase, respectively. * and ** indicate significance at 0.05 and 0.01 levels, respectively. C, P, T, C × P, C × T, P × T, and C × P × T refer to cultivar, phosphorus mode, temperature treatment, the interaction of cultivar with phosphorus mode, the interaction of cultivar with temperature treatment, the interaction of phosphorus mode with temperature treatment, and the interaction of cultivar, phosphorus mode with temperature treatment, respectively.

### Root osmotic regulatory substances contents

**Figure 3:**
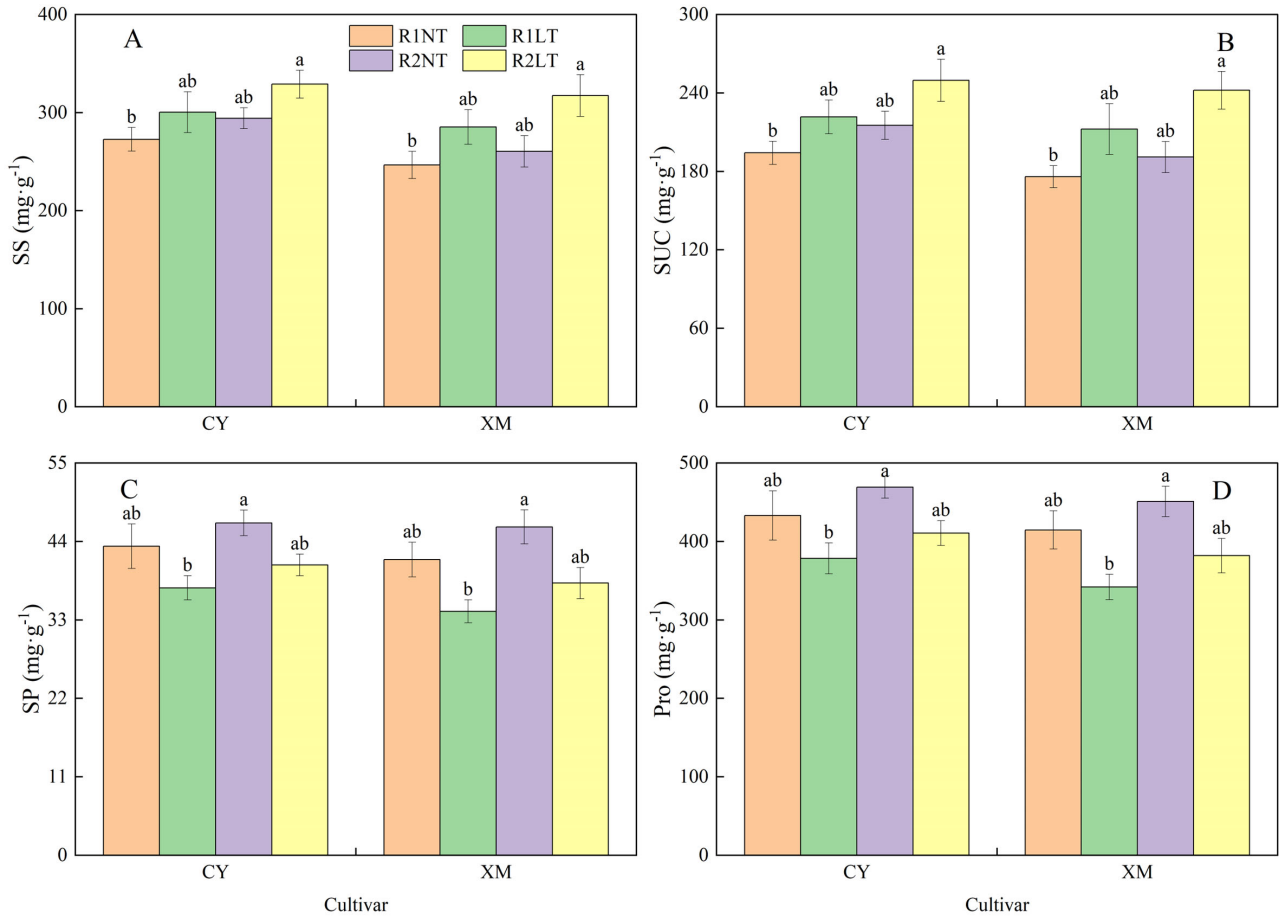
Effects of split-phosphorus application on osmotic regulatory substances contents in alfalfa roots under low-temperature stress. **(A)** Soluble sugar (SS) contents in alfalfa roots, **(B)** Sucrose concentrates (SUC) alfalfa roots, **(C)** Soluble protein (SP) alfalfa roots, and **(D)** Proline (Pro) contents in alfalfa roots. R1, R2, NT, and LT refer to traditional phosphorus application, split-phosphorus application, normal temperature, and low temperature, respectively. The data mean ± SE (*n* = 3). Lowercase letters refer to significant differences between the treatments (*P* < 0.05). Whiskers above the bars indicate the standard error.

The SS and SUC contents in the roots of CY and XM increased following LT stress, whereas SP and Pro contents decreased (**Figure 3**). Postponing phosphorus application increased the contents of SS, SUC, SP, and Pro alfalfa roots. The SS and SUC contents in the roots of CY and XM increased by 10.2-20.6% and 15.7-28.7%, 14.1-28.5% and 20.7-37.6%, respectively, under LT treatments. On the other hand, SP and Pro contents in the roots of CY and XM decreased by 6.0-13.4% and 7.9-17.6%, 5.2-12.6% and 7.9-17.5%, respectively. Under the same temperature treatments, postponing phosphorus application treatments exhibited an increasing trend for SS, SUC, SP, and Pro contents in the roots of CY and XM by 7.9-9.5% and 5.6-11.2%, 10.8-12.6% and 8.5-14.0%, 7.5-8.6% and 10.0-11.7%, 8.3-8.5% and 8.7-11.7%, respectively, compared to traditional phosphorus application treatments. Temperature had an extremely significant effect on osmotic regulatory substances contents in roots (*P* < 0.01), and phosphorus mode had a significant effect on the contents of SUC, SP, and Pro (*P* < 0.05), whereas cultivar had no significant effect on osmotic regulatory substances contents (**Table 3**).

### Root activity and ACP activity

**Figure 4:**
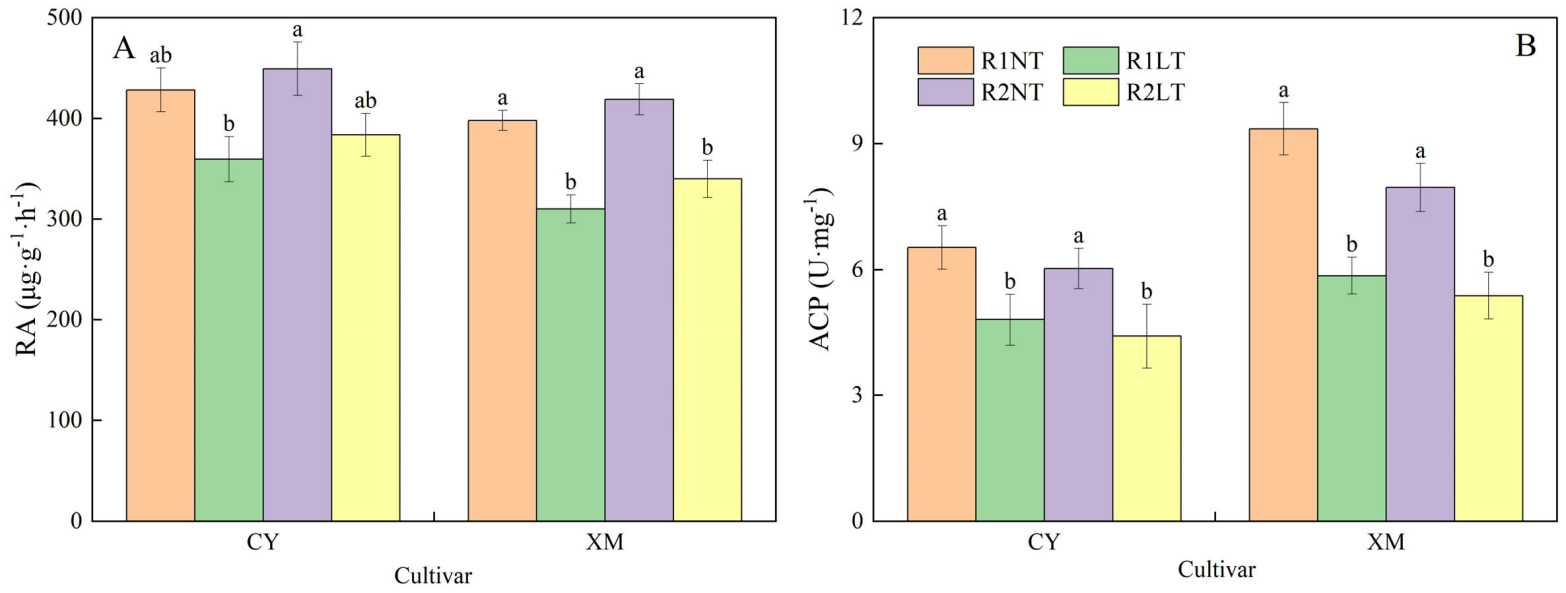
Effects of split-phosphorus application on root activities (RA) and acid phosphatase (ACP) activities in alfalfa roots under low-temperature stress. **(A)** RA in alfalfa roots, **(B)** ACP activities in alfalfa roots. R1, R2, NT, and LT refer to traditional phosphorus application, split-phosphorus application, normal temperature, and low temperature, respectively. The data mean ± SE (*n* = 3). Lowercase letters refer to significant differences between the treatments (*P* < 0.05). Whiskers above the bars indicate the standard error.

As shown in **Figure 4**, postponing phosphorus application treatments influenced root activities and ACP activities in alfalfa roots under LT stress. After LT stress, both root and ACP activities showed a decreasing trend, and ACP activities decreased significantly. The root activities of alfalfa increased after postponed phosphorus application, while the activities of ACP were further reduced. Specifically, CY and XM’s root and ACP activities decreased by 10.4-16.0% and 14.6-22.0%, 26.4-32.5%, and 38.4-44.4% under LT treatments, respectively. However, under the same temperature treatments, postponing phosphorus application treatments exhibited increased root activities in CY and XM by 4.9-6.7% and 5.3-9.6%, respectively, compared to traditional phosphorus application treatments, while ACP activities decreased by 7.7-8.2% and 9.7-11.1%, respectively. Temperature had an extremely significant effect on root activities and ACP activities in roots (*P* < 0.01), and cultivar had a significant effect on root activities (*P* < 0.05), whereas phosphorus mode had no significant effect on root activities and ACP activities (**Table 3**).

### Antioxidant enzymes activities and MDA contents in leaves

**Figure 5:**
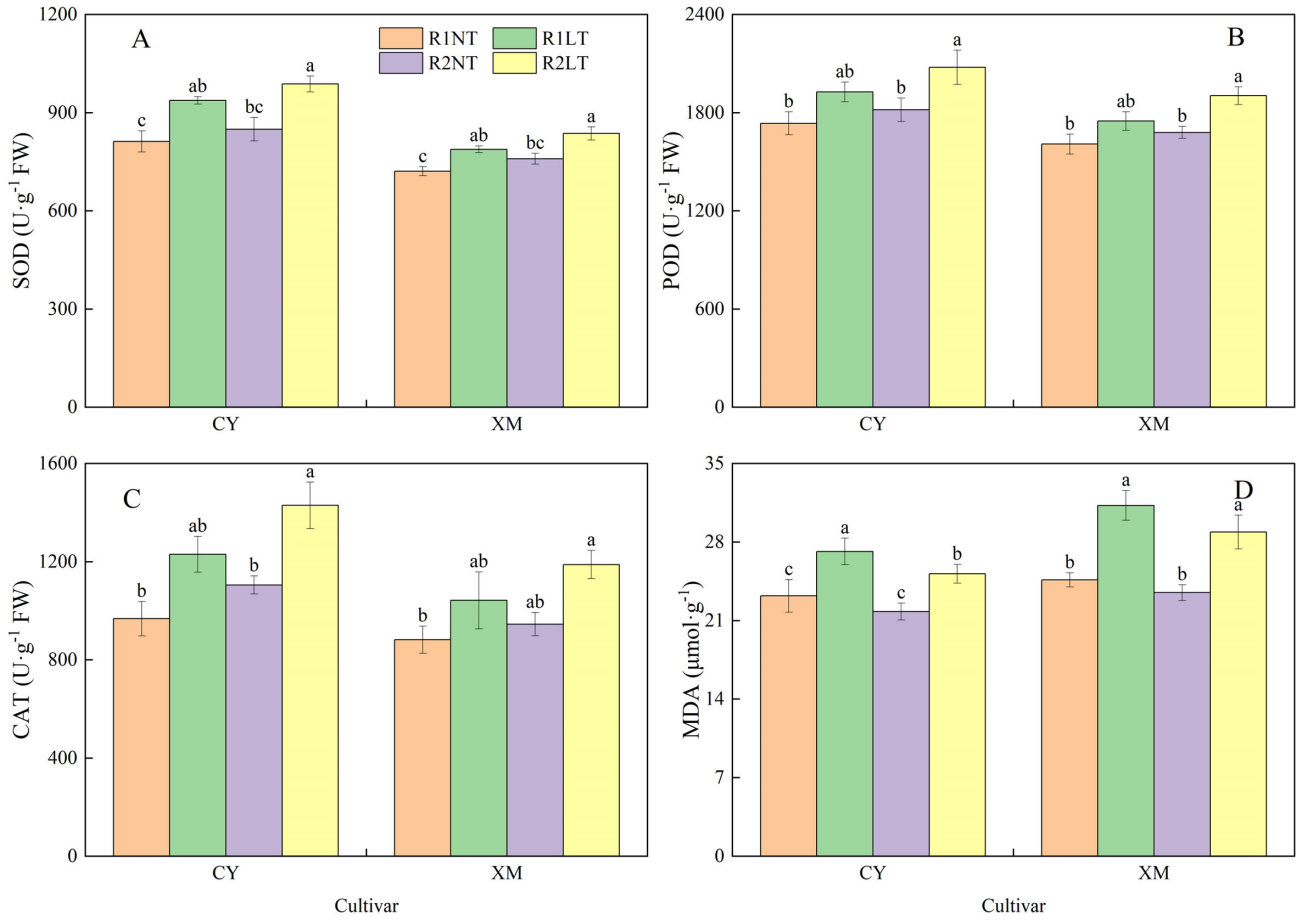
Effects of split-phosphorus application on antioxidant enzyme activities and malondialdehyde (MDA) constituents in alfalfa leaves under low-temperature stress. **(A)** Superoxide dismutase (SOD) activities in alfalfa leaves, **(B)** Peroxidase (POD) activities in alfalfa leaves, **(C)** Catalase (CAT) activities in alfalfa leaves, and **(D)** MDA contents in alfalfa leaves. R1, R2, NT, and LT refer to traditional phosphorus application, split-phosphorus application, normal temperature, and low temperature, respectively. The data mean ± SE (*n* = 3). Lowercase letters refer to significant differences between the treatments (*P* < 0.05). Whiskers above the bars indicate the standard error.

**Figure 5** illustrates the activities of SOD, POD, and CAT, as well as MDA contents in leaves of CY and XM cultivars, similar to the changes observed in the roots (**Figure 2**). The activities of SOD, POD, and CAT in the leaves of CY and XM increased by 15.4-21.6% and 9.2-16.0%, 11.1-19.8% and 8.8-18.4%, 27.1-47.7% and 18.1-34.7%, respectively, after LT treatment. Concurrently, the MDA contents in leaves of both CY and XM significantly increased by 8.5-17.1% and 17.3-26.9%, respectively, following the LT treatments. When subjected to split-phosphorus application treatments at the same temperatures, the SOD, POD, and CAT activities in leaves of CY and XM increased by 4.6-5.3% and 5.2-6.2%, 11.2-19.8% and 8.8-18.4%, 14.2-16.2% and 7.2-14.0%, respectively, compared to the traditional phosphorus application treatments. Furthermore, the MDA contents in roots of CY and XM under split-phosphorus application treatments decreased by 6.0-7.4% and 4.6-7.6%, respectively, and a significant reduction was observed in the MDA contents of CY under R2LT treatment compared to R1LT treatment. Cultivar and temperature had an extremely significant effect on antioxidant enzyme activities and MDA contents in leaves (*P* < 0.01), whereas phosphorus mode had a significant effect on the activities of SOD, POD, and CAT (*P* < 0.05; **Table 4**).

**Table 4:** Three-way ANOVA analysis for the effects of split-phosphorus application on antioxidant enzymes activities, MDA contents, and osmotic regulatory substances in alfalfa leaves under low-temperature stress and the interactive effect.

F-value	SOD	POD	CAT	MDA	SS	SUC	SP	Pro
C	52.676**	11.794**	9.567**	26.477**	5.539*	3.406	7.958*	3.213
P	6.829*	6.577*	6.266*	33.802**	6.945*	5.357*	4.215	4.372
T	37.629**	20.797**	20.572**	124.544**	27.620**	20.340**	18.981**	18.222**
C×P	0	0.002	0.34	4.29	0.001	0.019	0.002	0.005
C×T	3.206	0.23	0.71	0.22	0.14	0.434	0.162	0.297
P×T	0.127	0.712	0.44	1.29	0.597	0.365	0.096	0.004
C×P×T	0.001	0.009	0.01	1.97	0.02	0.055	0.001	0.005

Here, SOD, POD, CAT, MDA, SS, SUC, SP, and Pro refer to superoxide dismutase, peroxidase, catalase, malondialdehyde, soluble sugar, sucrose, soluble protein, and proline, respectively. * and ** indicate significance at 0.05 and 0.01 levels, respectively. C, P, T, C × P, C × T, P × T, and C × P × T refer to cultivar, phosphorus mode, temperature treatment, the interaction of cultivar with phosphorus mode, the interaction of cultivar with temperature treatment, the interaction of phosphorus mode with temperature treatment, and the interaction of cultivar, phosphorus mode with temperature treatment, respectively.

### Osmotic adjustment substances in leaves

**Figure 6:**
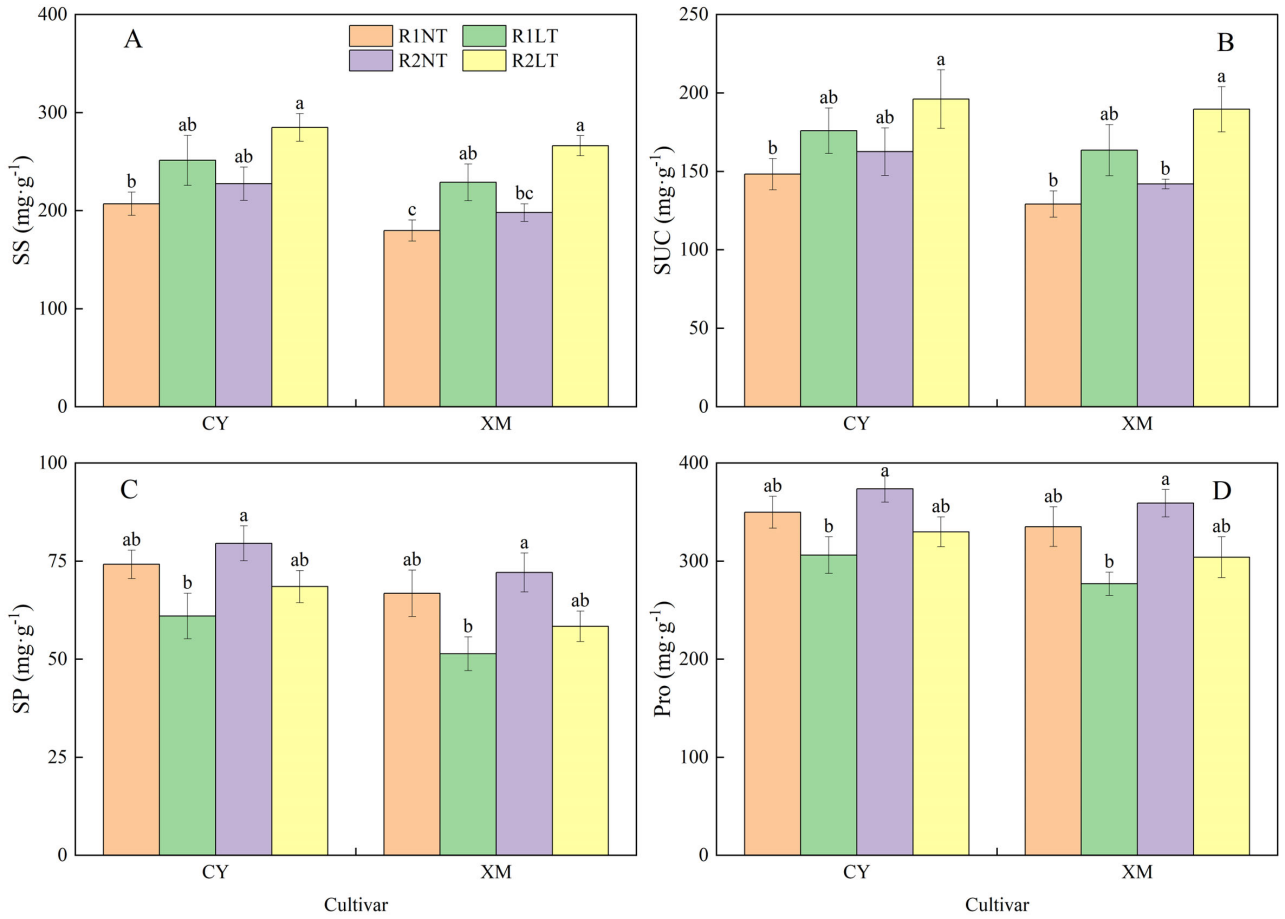
Effects of split-phosphorus application on osmotic regulatory substances in alfalfa leaves under low-temperature stress. **(A)** Soluble sugar contents in alfalfa leaves, **(B)** Sucrose contents in alfalfa leaves, **(C)** Soluble protein in alfalfa leaves, and **(D)** Proline contents in alfalfa leaves. R1, R2, NT, and LT refer to traditional phosphorus application, split-phosphorus application, normal temperature, and low temperature, respectively. The data mean ± SE (*n* = 3). Lowercase letters refer to significant differences between the treatments (*P* < 0.05). Whiskers above the bars indicate the standard error.

As shown in **Figure 6**, the SS and SUC contents in leaves of CY and XM increased after LT stress, while SP and Pro contents decreased. Under postponing phosphorus application treatments, the SS, SUC, SP, and Pro contents in alfalfa leaves increased. Particularly, the SS and SUC contents in leaves of CY and XM increased by 21.4-37.6% and 27.3-48.0%, 18.6-32.3%, and 26.6-46.8%, respectively, under LT treatments. Concurrently, the contents of SP and Pro in leaves of CY and XM decreased by 7.6-17.8% and 12.6-23.1%, 5.7-12.5%, and 9.3-17.4%, respectively, under LT treatments. Under the same temperature treatment, the contents of SS, SUC, SP, and Pro in leaves of CY and XM increased by 9.9-13.4% and 10.2-16.3%, 9.7-11.5% and 9.9-16.0%, 7.2-12.3% and 7.9-13.6%, 6.8-7.7% and 7.1-9.8% under the treatments of postponing phosphorus application, respectively, compared to the traditional-phosphorus application. Temperature had an extremely significant effect on osmotic regulatory substances contents in leaves (*P* < 0.01), cultivar had a significant effect on the contents of SS and SP (*P* < 0.05), and phosphorus mode had a significant effect on the contents of SS and SUC (**Table 4**).

### Carbon and nitrogen metabolism-related enzymatic activities in leaves

**Figure 7:**
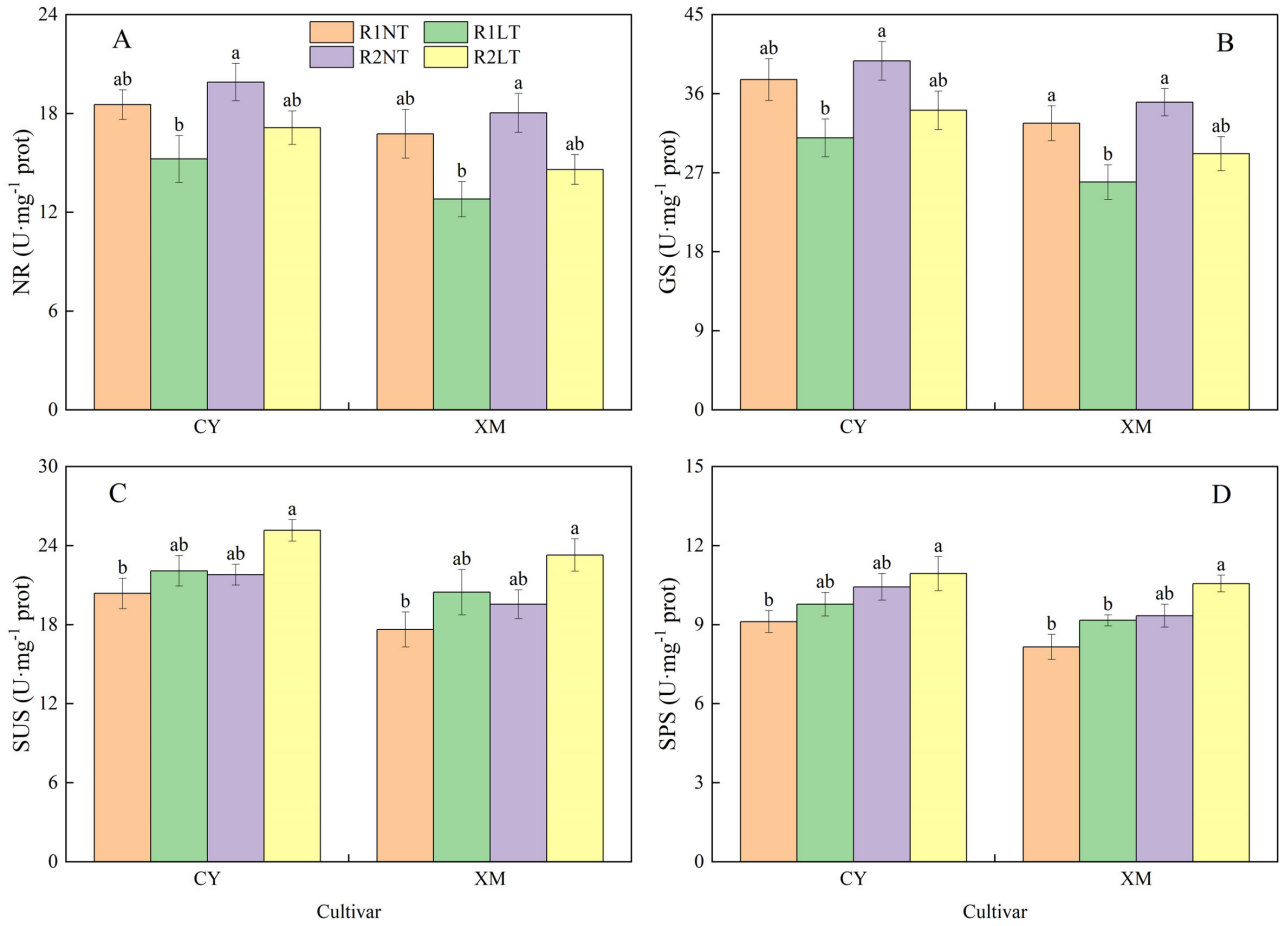
Effects of split-phosphorus application on carbon and nitrogen metabolism-related enzyme activities in alfalfa leaves under low-temperature stress. **(A)** Nitrate reductase (NR) activities in alfalfa leaves, **(B)** Glutamine synthetase (GS) activities in alfalfa leaves, **(C)** Sucrose synthase (SUS) activities in alfalfa leaves, and **(D)** Sucrose phosphate synthase (SPS) activities in alfalfa leaves. R1, R2, NT, and LT refer to traditional phosphorus application, split-phosphorus application, normal temperature, and low temperature, respectively. The data mean ± SE (*n* = 3). Lowercase letters refer to significant differences between the treatments (*P* < 0.05). Whiskers above the bars indicate the standard error.

The enzymatic activities of carbon and nitrogen metabolism in leaves exhibited a similar trend to the changes in osmotic adjustment substances (**Figures 6**, **7**). The NR and GS activities in the leaves of CY and XM decreased after LT stress, while the SUS and SPS activities increased. The postponed phosphorus application increased in alfalfa leaves’ NR, GS, SUS, and SPS activities. The NR and GS activities in leaves of CY and XM decreased by 7.6-17.8% and 12.9-23.7%, 9.3-17.6% and 10.6-20.5%, respectively, after LT treatments. On the other hand, the SUS and SPS activities increased by 8.4-23.5%, 16.0-31.9%, 7.2-20.0%, and 12.4-29.5%, respectively, after LT treatments. Under the same temperature treatments, the NR, GS, SUS, and SPS activities in leaves of CY and XM increased by 7.4-12.5% and 7.6-14.1%, 5.7-10.1% and 7.4-12.5%, 7.0-13.9% and 10.8-13.7%, 11.9-14.4% and 14.6-15.2%, respectively, under postponing phosphorus application treatments compared to the traditional phosphorus application treatments. Temperature had an extremely significant effect on the enzymatic activities of carbon and nitrogen metabolism in leaves (*P* < 0.01), cultivar had a significant effect on them (*P* < 0.05), while phosphorus mode had no significant effect on the enzymatic activities of NR and GS (**Table 5**).

**Table 5:** Three-way ANOVA analysis for the effects of split-phosphorus application on carbon and nitrogen metabolism-related enzymatic activities, SPAD values, chlorophyll contents, and photosynthetic parameters in alfalfa leaves under low-temperature stress, and the interactive effect.

F-value	NR	GS	SUS	SPS	SPAD	Chl	Pn	Gs	Ci	Tr
C	7.906*	13.597**	5.741*	7.585*	11.024**	40.153**	3.322	32.162**	13.730**	3.582
P	4.287	4.191	6.798*	20.923**	5.889*	7.333*	3.322	17.704**	53.522**	3.37
T	19.385**	21.753**	10.853**	9.410**	20.499**	32.472**	27.066**	48.837**	174.330**	23.071**
C×P	0.004	0.005	0.00	0.01	0.053	0.019	0.027	0.301	0.019	0.138
C×T	0.19	0.003	0.18	0.93	0	2.263	0.247	0.108	0.89	0.048
P×T	0.122	0.119	0.52	0.00	0.159	0.126	0.001	0.301	1.764	0.194
C×P×T	0	0.001	0.04	0.11	0.042	0.07	0.001	0.012	0.128	0.076

NR, GS, SUS, SPS, SPAD, Chl, Pn, Gs, Ci, and Tr refer to nitrate reductase, glutamine synthetase, sucrose synthase, sucrose phosphate synthase, SPAD value, chlorophyll content, net photosynthetic rate, stomatal conductance, intercellular CO_2_ concentration, and transpiration rate, respectively. * and ** indicate significance at 0.05 and 0.01 levels, respectively. C, P, T, C × P, C × T, P × T, and C × P × T refer to cultivar, phosphorus mode, temperature treatment, the interaction of cultivar with phosphorus mode, the interaction of cultivar with temperature treatment, the interaction of phosphorus mode with temperature treatment, and the interaction of cultivar, phosphorus mode with temperature treatment, respectively.

### SPAD values and chlorophyll contents in the leaves

**Figure 8:**
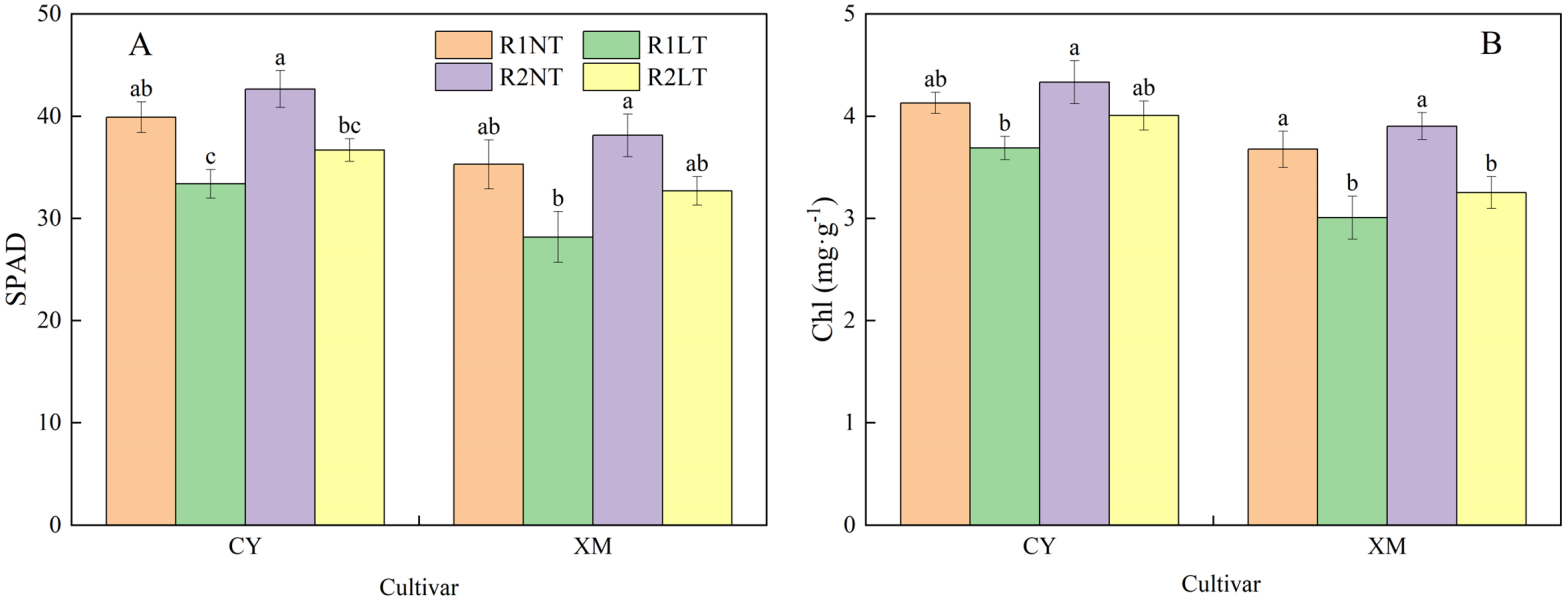
Effects of split-phosphorus application on SPAD values (SPAD) and chlorophyll (Chl) content in alfalfa leaves under low-temperature stress. **(A)** SPAD in alfalfa leaves, **(B)** Chl content in alfalfa leaves. R1, R2, NT, and LT refer to traditional phosphorus application, split-phosphorus application, normal temperature, and low temperature, respectively. The data mean ± SE (*n* = 3). Lowercase letters refer to significant differences between the treatments (*P* < 0.05). Whiskers above the bars indicate the standard error.

The effects of split-phosphorus application on SPAD values and Chl contents of alfalfa leaves under LT stress are illustrated in **Figure 8**. The SPAD values and Chl contents in the leaves of CY and XM decreased after LT stress; a significant reduction was observed in the Chl contents of XM leaves. Postponing phosphorus application resulted in varying degrees of increase in both indicators for alfalfa leaves. The SPAD values and Chl contents of CY and XM leaves declined by 8.0-16.3% and 7.4-20.1%, 3.0-10.7%, and 11.5-18.1%, respectively, under LT treatment. However, under the same temperature treatments, the SPAD values and Chl contents of CY and XM leaves after postponing phosphorus application increased by 6.9-9.9% and 8.0-16.0%, 4.8-8.6% and 6.2-8.1%, respectively, compared to the traditional-phosphorus application treatments. Temperature and cultivar had an extremely significant effect on SPAD values and chlorophyll content in leaves (*P* < 0.01), and phosphorus mode had a significant effect on SPAD values and chlorophyll content (*P* < 0.05; **Table 5**).

### Leaf photosynthetic parameters

**Figure 9:**
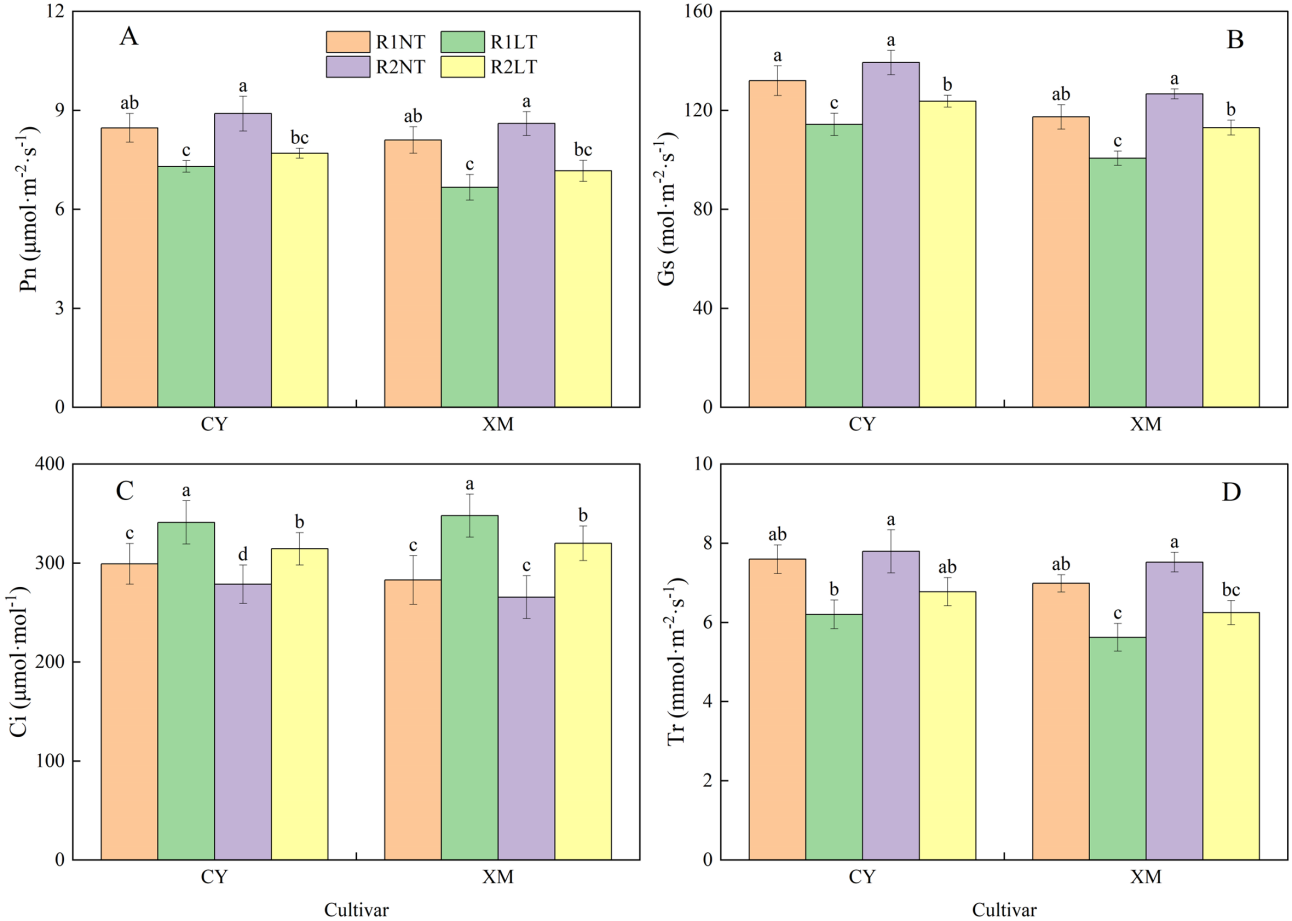
Effects of split-phosphorus application on photosynthetic parameters in alfalfa leaves under low-temperature stress. **(A)** Net photosynthetic rate (Pn) in alfalfa leaves, **(B)** Stomatal conductance (Gs) in alfalfa leaves, **(C)** Intercellular carbon-dioxide concentration (Ci) in alfalfa leaves, and **(D)** Transpiration rate (Tr) in alfalfa leaves. R1, R2, NT, and LT refer to traditional phosphorus application, split-phosphorus application, normal temperature, and low temperature, respectively. The data mean ± SE (*n* = 3). Lowercase letters refer to significant differences between the treatments (*P* < 0.05). Whiskers above the bars indicate the standard error.

**Figure 9** illustrates the influence of split-phosphorus application on the photosynthesis parameters of CY and XM leaves under LT stress. The increasing trend was found in Ci, opposite Pn, Gs, and Tr. The Pn, Gs, and Tr of CY and XM leave decreased by 9.1-13.8% and 11.5-17.7%, 6.3-13.4% and 3.7-14.2%, 10.8-18.3% and 10.6-19.5%, respectively, after LT treatment. Meanwhile, Ci increased significantly by 5.1-14.0% and 7.8-17.7% in CY and XM leaves under LT treatments. Under the same temperature conditions, Pn, Gs, and Tr of CY and XM leaves increased by 5.1-5.5% and 6.2-7.5%, 5.6-8.2% and 8.0-12.3%, 2.6-9.2% and 7.7-11.1%, respectively, with postponing phosphorus application compared to the traditional phosphorus application treatments. On the other hand, Ci decreased by 6.9-7.8% and 6.1-8.4% in CY and XM leaves under postponed phosphorus application treatments. Moreover, the Ci of R2LT treatment was significantly lower than that of R1LT in both cultivars. Temperature had an extremely significant effect on the photosynthetic parameters in leaves (*P* < 0.01), and cultivar and phosphorus mode had a significant effect on Gs and Ci (*P* < 0.05), while cultivar and phosphorus mode had no significant effect on Pn and Tr (**Table 5**).

### Correlation analysis between physiological indicators of alfalfa leaves with plant dry weight, root dry weight, and phosphorus

**Figure 10:**
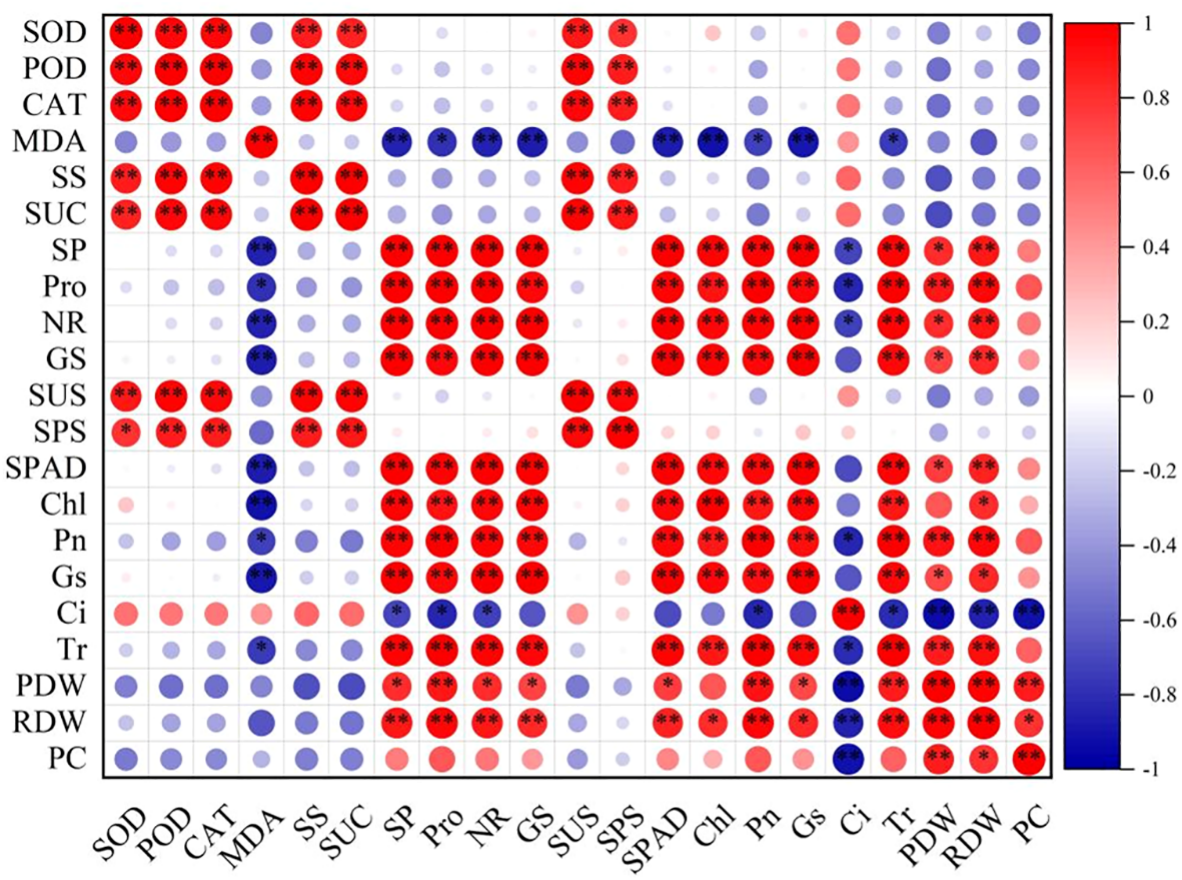
Correlation analysis between physiological indicators of alfalfa leaves with plant dry weight, root dry weight and plant phosphorus concentration. * and ** indicate significance at 0.05 and 0.01 levels, respectively. SOD, POD, CAT, MDA, SS, SUC, SP, Pro, NR, GS, SUS, SPS, SPAD, Chl, Pn, Gs, Ci, Tr, PDW, RDW, and PC refer to superoxide dismutase, peroxidase, catalase, malondialdehyde, soluble sugar, sucrose, soluble protein, proline, nitrate reductase, glutamine synthetase, sucrose synthase, sucrose phosphate synthase, SPAD value, chlorophyll content, net photosynthetic rate, stomatal conductance, intercellular CO_2_ concentration, transpiration rate, plant dry weight, root dry weight and plant phosphorus concentration, respectively.

Correlation analysis between physiological indicators of alfalfa leaves with plant dry weight, root dry weight and plant phosphorus concentration is presented in **Figure 10**. There are significant (*P* < 0.05) or extremely significant (*P* < 0.01) positive correlations between plant dry weight, root dry weight with SP, Pro, NR, GS, SPAD, Pn, Gs, and Tr, but significantly negative (*P* < 0.01) correlations with Ci. The PC is extremely significant positive correlations with PDW and extremely significant (*P* < 0.01) negatively correlated with Ci. These results indicate that plant dry weight, root dry weight are largely determined by the photosynthetic capacity and osmotic adjustment substances.

## Discussion

### Effects of split-phosphorus application on antioxidants and acid phosphatase activities in Alfalfa under low-temperature stress

The production of ROS is an inevitable metabolic byproduct during plant growth and development. Under normal conditions, plants utilize their protective enzyme system (containing SOD, POD, CAT, etc.) to eliminate these ROS from plant cells (Caverzan et al., 2016; Liu et al., 2019). However, when plants are subjected to LT stress, their ability to utilize and decompose ROS significantly decreases, leading to excessive accumulation of ROS within the plant cells. Under LT stress, lipid peroxidation occurs, leading to the excessive accumulation of MDA and causing oxidative damage to the plant cell’s membranous structure; in severe cases, cell necrosis occurs (Su et al., 2022). When plants are exposed to LT stress, plant cells sense stress signals through their cell membranes and transmit these signals to activate their innate antioxidant enzymatic system to eradicate excessive ROS and prevent lipid peroxidation, thus protecting the plant from harmful impacts of LT (Zhang et al., 2022). This regulatory mechanism is more visible in cultivars with higher LT tolerance, as they exhibit stronger protective enzymatic activities (Chen et al., 2015). In this study, the antioxidant enzyme activities in leaves and roots of the alfalfa cultivar CY were higher than those of XM after LT stress. Lower MDA contents potentially explain the higher LT tolerance of CY. In addition to enhancing the antioxidant enzyme system, alfalfa also reduces ROS production by adjusting metabolic pathways or improving ROS scavenging ability by synthesizing more antioxidants (Chen et al., 2015). Some studies have shown that exogenous application of certain chemicals or optimizing water and fertilizer management can also improve the resistance of alfalfa to LT stress (Li et al., 2022; Zhang et al., 2023). Our findings revealed that the activities of SOD, POD, and CAT in the roots of the two cultivars increased by 6.3-13.1%, 7.8-11.4%, and 5.1-13.3%, respectively, under postponing phosphorus application, compared to traditional phosphorus application treatments. Similarly, SOD, POD, and CAT activities in the leaves increased by 4.6-6.2%, 8.8-19.8%, and 7.2-16.2%, while MDA content decreased in both cases. These results are consistent with previous studies, indicating that appropriate phosphorus fertilization can enhance the antioxidant enzymes’ activities and reduce alfalfa plants’ oxidative-damaging substances’ content (Nasar et al., 2022; Li et al., 2020b). Xia et al. (2022) also noted that phosphorus application increased SOD activity in alfalfa root crown by 26.4-35.6% and reduced MDA content by 48.6-50.9% under LT stress.

As a vital hydrolase in plants, ACP plays an essential role in plant C-N metabolism and decomposes and reuses phosphorus (Yang and Yang, 2021). The plant’s phosphorus supply level affects the activity of ACP (Widdig et al., 2019). When plants undergo stress caused by phosphorus deficiency, the activity of ACP increases significantly (Dissanayaka et al., 2021). This study showed that the ACP activities of alfalfa roots decreased significantly by 26.4-44.4% under LT stress, and successive phosphorus applications also reduced the ACP activities. LT stress decreased the activity of ACP in roots, which may be due to the decrease of enzyme activity directly caused by abiotic stress. On the other hand, the ACP activities decreased further due to the sufficient phosphorus availability for alfalfa roots (Xu et al., 2023b).

### Effects of split-phosphorus application on physiology of carbon and nitrogen metabolism in alfalfa under low-temperature stress

Carbon and nitrogen metabolism are vital physiological processes for plant growth and development, and they are interdependent. Nitrogen metabolism provides enzymes and photosynthetic pigments for carbon metabolism, while carbon metabolism supplies carbon compounds and energy sources for nitrogen metabolism (Baslam et al., 2020; Huppe and Turpin, 1994). Both physiological processes required a standard reducing power, ATP, and carbon skeletons. LT stress inhibits respiratory and photosynthetic processes, impairing carbon metabolism and impeding nitrogen absorption and plant utilization. In our study, under LT stress, the activities of carbon metabolism-related enzymes and the contents of related substances in the roots and leaves of alfalfa increased to varying degrees compared to the normal temperature conditions. On the contrary, the activities of nitrogen metabolism-related enzymes and the contents of related substances decreased. These results are coherent with the findings of (Zhu et al., 2015), indicating that LT stress induces an increase in plant carbon metabolism enzymes and substances to adapt to stressful environmental conditions while inhibiting nitrogen metabolism physiological processes.

Following the postponement of phosphorus application, the activities of carbon and nitrogen metabolism-related enzymes and the contents of related substances increased in alfalfa leaves and roots. The activities of NR, GS, SUS, and SPS in the leaves increased by 7.4-14.1%, 5.7-12.5%, 7.0-13.9%, and 11.9-15.2%, respectively, compared to traditional phosphorus application treatments. Additionally, SS, SUC, SP, and Pro activities in the leaves increased by 5.6-11.2%, 8.5-14.0%, 7.5-11.7%, and 8.3-11.7%, respectively. These findings suggested that postponing phosphorus application enhances the activities of enzymes related to carbon and nitrogen metabolism in the synthetic direction, increasing the contents of osmotic regulatory substances, thus maintaining osmotic balance in alfalfa plants. Previous studies have indicated that phosphorus enhances the nitrogen metabolism of alfalfa by activating nitrate reductase and glutamine synthetase enzymes (Yu et al., 2018), which is consistent with our findings. Moreover, optimizing phosphorus application promotes the accumulation of carbohydrates and nitrogen-containing protective sub-stances in alfalfa plants, thereby improving their LT tolerance capacity. Under LT treatment, postponing phosphorus application increased soluble sugar and sucrose contents in alfalfa root crowns by 2.1-26.9% and 33.5-84.6%, respectively, compared to traditional phosphorus application treatments, enhancing root crown vitality and LT tolerance (Xia et al., 2022). These results suggested that postponing phosphorus application improves carbon and nitrogen metabolism in alfalfa plants, increasing the accumulation of osmotic regulatory substances and enhancing the plant’s LT tolerance.

### Effects of split-phosphorus application on photosynthetic pigments and characteristics in alfalfa under low-temperature stress

Photosynthetic pigments and parameters are crucial to alfalfa’s growth, yield, and environmental responsiveness (Xu et al., 2020) Photosynthetic pigments are pivotal in absorbing, transferring light energy, or initiating primary photochemical reactions (Simkin et al., 2022). Under LT stress, the Chl contents decrease in alfalfa leaves. LT tolerant cultivar CY showed a 3.0-10.7% reduction in Chl contents, while the LT sensitive cultivar XM exhibited a significant 11.5-18.1% decrease in Chl contents. These results may be attributed to the damage induced to the thylakoid membrane structure, affecting its permeability and fluidity, thus hampering typical chloroplast functioning (Liu et al., 2018; Venzhik et al., 2016). The intensity of the damage to sensitive cultivars was higher than that of tolerant cultivars.

The reduction in photosynthetic pigment contents directly impacts photosynthesis by diminishing the plant’s ability to capture light energy (Mussgnug et al., 2007; Simkin et al., 2022). Previous research studies have also revealed the negative impacts of LT stress on photosynthetic pigment synthesis and photosynthesis in alfalfa. At the same time, silicon maintains the stability of photosystem II and photosynthetic pigments for sustaining normal plant growth (Rahman et al., 2024). We observed that the Pn of leaves in both cultivars, CY and XM, decreased by 9.1-13.8% and 11.5-17.7%, respectively, after LT stress. The increase in Ci after LT stress suggested that non-stomatal factors primarily limit the photosynthetic capacity. Phosphorus, a crucial nutrient for chlorophyll synthesis, promotes its production and increases its contents. Optimizing phosphorus application has improved water use efficiency, leaf Pn, reduced damage to photosynthetic organs, and enhanced alfalfa’s stress tolerance (Xia et al., 2023). In this study, postponing phosphorus application increased Chl contents in alfalfa leaves by 4.8-8.6% compared to traditional phosphorus application treatments. Chl is an essential biochemical substance for capturing solar energy and completing the photo-synthetic process by facilitating the increase in Pn, Gs, and Tr in CY and XM under postponing phosphorus application by 5.1-5.5% and 6.2-7.5%, 5.6-8.2% and 8.0-12.3%, and 2.6-9.2% and 7.7-11.1%, respectively. Phosphorus promotes ATP synthesis and improves cell membrane permeability and enzyme activity, thereby accelerating the transport of photosynthetic products and enhancing net photosynthetic rates (Balemi and Negisho, 2012; Xu et al., 2023b). The increase in Gs and Tr helps to maintain gaseous exchange between alfalfa leaves and the atmosphere, ensuring smooth photosynthesis (Jiang et al., 2009; Mashilo et al., 2017). In brief, optimizing phosphorus application affects the photosynthetic pigment contents in alfalfa leaves and indirectly influences photosynthesis by improving soil conditions and promoting root development. These combined effects enhance alfalfa’s tolerance and productivity under LT stress.

## Conclusion

We comprehensively analyzed the relevant physiological parameters of two alfalfa cultivars with different LT tolerances to investigate the mitigating effects of postponed phosphorus application on alfalfa under LT stress. Our findings reveal that LT stress significantly contributes to the accumulation of membrane lipid peroxide MDA in plants, causing more significant damage to the LT-sensitive cultivar XM. However, postponing phosphorus application enhances alfalfa cultivars’ antioxidant enzyme activities, car-bon-nitrogen metabolism-related enzyme activities, and metabolite contents. This enhancement subsequently improves the osmotic adjustment balance and photosynthetic productivity, favoring alfalfa’s normal growth, development, and quality enhancement. Future research will investigate the molecular mechanisms influencing alfalfa’s growth and development. Simultaneously, we will explore optimizing water and fertilizer management practices in production settings, providing valuable support for producing high-quality alfalfa and stress resistance.

## Data Availability

The original contributions presented in the study are included in the article/supplementary material. Further inquiries can be directed to the corresponding authors.
